# Revealing the significance of IL-2 and IL-5 in SARS-CoV-2-specific T-cell responses in kidney transplant recipients

**DOI:** 10.1038/s44298-024-00015-7

**Published:** 2024-02-14

**Authors:** Yvette den Hartog, S. Reshwan K. Malahe, Wim J. R. Rietdijk, Marjolein Dieterich, Lennert Gommers, Debbie van Baarle, Dimitri A. Diavatopoulos, A. Lianne Messchendorp, Renate G. van der Molen, Ester B. M. Remmerswaal, Frederike J. Bemelman, Marcia M. L. Kho, Corine H. GeurtsvanKessel, Marion P. G. Koopmans, Ron T. Gansevoort, Luuk B. Hilbrands, Jan-Stephan Sanders, Marlies E. J. Reinders, Carla C. Baan, Rory D. de Vries, on behalf of RECOVAC Consortium, Alferso C. Abrahams, Marije C. Baas, Pim Bouwmans, Marc H. Hemmelder, Marc A. G. J. ten Dam, Sophie C. Frölke, Dorien Standaar, Marieke van der Heiden, Celine Imhof, Priya Vart, Yvonne M. R. Adema, Marieken J. Boer-Verschragen, Wouter B. Mattheussens, Ria Philipsen, Djenolan van Mourik, Nynke Rots, Gerco den Hartog, Rob van Binnendijk

**Affiliations:** 1 Department of Internal Medicine, Nephrology and Transplantation, Erasmus MC Transplant Institute, University Medical Center Rotterdam, Rotterdam, The Netherlands; 2 Department of Hospital Pharmacy, Erasmus MC, University Medical Center Rotterdam, Rotterdam, The Netherlands; 3 Department of Viroscience, Erasmus MC, University Medical Center Rotterdam, Rotterdam, The Netherlands; 4 Department of Medical Microbiology and Infection Prevention, University Medical Center Groningen, Groningen, The Netherlands; 5 Center for Infectious Disease Control, National Institute for Public Health and the Environment, Bilthoven, The Netherlands; 6 Department of Laboratory Medicine, Laboratory of Medical Immunology, Radboud University Medical Center Nijmegen, Nijmegen, The Netherlands; 7 Radboud Center for Infectious Diseases, Radboud University Medical Center Nijmegen, Nijmegen, The Netherlands; 8 Department of Internal Medicine, Division of Nephrology, University of Groningen, University Medical Center Groningen, Groningen, the Netherlands; 9 Department of Experimental Immunology, Amsterdam Infection and Immunity Institute, Amsterdam UMC, University of Amsterdam, Amsterdam, The Netherlands; 10 Renal Transplant Unit, Amsterdam UMC, University of Amsterdam, Amsterdam, The Netherlands; 11 Department of Nephrology, Radboud University Medical Center Nijmegen, Nijmegen, The Netherlands; 12 Department of Nephrology and Hypertension, University Medical Center Utrecht, Utrecht, The Netherlands; 13 Department of Internal Medicine, division of Nephrology, Maastricht University Medical Center and CARIM school for cardiovascular disease, University of Maastricht, Maastricht, The Netherlands; 14 Dutch Registry RENINE, Nefrovisie, Utrecht, The Netherlands

**Keywords:** Immunological memory, Antibodies, RNA vaccines

## Abstract

Kidney transplant recipients (KTRs) are at an increased risk of severe COVID-19 due to compromised immune responses. Although vaccination is critical in preventing severe disease, KTRs have attenuated vaccination-induced immune responses due to underlying kidney disease and immunosuppressive therapies. In this study, the effect of different COVID-19 booster strategies on SARS-CoV-2-specific T-cell responses was assessed in KTRs who showed a poor serological response after the first two mRNA-based primary vaccination doses. In these KTRs, a third vaccination dose led to an increase in antibody levels in the majority of patients. Production of IL-2 and IL-5 by SARS-CoV-2 specific T cells positively correlated with antibody levels, with stronger correlations compared to IFN-γ production, the ‘traditional’ cytokine to measure T-cell responses. Our study underscores the significance a balanced T-cell cytokine response to achieve robust antibody responses in KTRs. Furthermore, we show that multiple cytokines to assess T-cell responses should be explored to identify individuals in need of tailored vaccination strategies.

## Introduction

Kidney transplant recipients (KTRs) are at increased risk of severe outcomes associated with coronavirus disease-2019 (COVID-19)^1^. This was most pronounced in the initial stages of the pandemic, when vaccines were not yet readily available^2–5^. However, with the introduction of vaccination for pandemic control, there has been a substantial reduction in fatal disease progression and mortality rates observed in KTRs. Nevertheless, KTRs continue to face a higher risk compared to immunocompetent individuals^3,6–8^.

Unlike immunocompetent individuals, KTRs often do not mount effective immune responses after vaccination, primarily due to immunosuppressive therapies^5,9–13^. In the context of COVID-19, KTRs had compromised humoral and cellular immune responses compared to the general population after completion of primary vaccination with mRNA-based COVID-19 vaccines. Antibody production was particularly affected, with significantly lower levels than observed in the general population^9,10,12^. T-cell responses were also affected, which is important as T-cells play a vital role in guiding the maturation and differentiation of B-cells. When activated, T-cells secrete various cytokines, orchestrating an environment critical for B-cell differentiation into plasma cells^14–19^. In previous research, we demonstrated that achieving a balanced T-helper (Th)1 / Th2 cytokine profile by mRNA-1273 COVID-19 vaccination is important for antibody production^14^.

Recently, we showed that despite initial poor responses to the first two vaccinations, administration of additional vaccines to KTRs can boost severe acute respiratory syndrome coronavirus-2 (SARS-CoV-2)-specific antibody responses^20^. We boosted KTRs with a single dose of mRNA-1273, a double dose of mRNA-1273, or a single dose of Ad26.COV2.S, and observed that the three strategies were equally immunogenic. Whether repeated vaccination enhanced T-cell responses remained unclear, as an increase in SARS-CoV-2-specific T-cells could not be detected by interferon (IFN)-γ ELISPOT, but was detected by IFN-γ release assay (IGRA)^20^. Different results obtained with these assays could be explained by the fact that the IFN-γ ELISPOT is performed with peripheral blood mononuclear cells (PBMC), whereas the IGRA is performed in whole blood. Performing IGRA to measure virus-specific T-cell responses may be more reliable for this specific patient group, as the assay is performed in a physiologically relevant environment, i.e. in the presence of immunosuppressive drugs^21^.

In the subsequent phases of the COVID-19 outbreak, the ongoing evolution of SARS-CoV-2 presented persistent challenges, as antigenic changes led to the evasion of antibodies induced against the ancestral viral spike (S) proteins^22^, potentially making the role of T-cells reactive with conserved epitopes in the S protein even more important. Updated vaccines, initially bivalent and currently monovalent, are recommended for KTRs, attempting to redirect the immune response to distinct variants to maintain immunity against antigenically distinct SARS-CoV-2 variants. Although it is unclear whether booster vaccination of KTRs increases T-cell responses, it is encouraging that SARS-CoV-2-specific CD4 and CD8 T-cells induced by initial vaccination cross-recognize novel variants^23,24^. Furthermore, these T-cells have been associated with protection and early recovery from COVID-19, even in the absence of robust humoral responses^25–28^. This emphasizes the critical need for their detection and gaining more insight into T-cell responses, particularly when studying vaccine immunogenicity in immunocompromised individuals.

In this study, we investigated T-cell cytokine responses and their potential correlation with antibody responses in KTRs who had a poor serological response to two primary mRNA-1273 vaccinations. These KTRs were randomly assigned to receive a booster with either a double dose of mRNA-1273, a heterologous vaccination with Ad26.COV2.S, or a single dose of mRNA-1273. We evaluated S-specific antibody responses and T-cell cytokine profiles before and 28 days after booster vaccination. These analyses aimed to reveal the association of T-cell cytokine diversity on the SARS-CoV-2 antibody response and shed light on the significance of specific cytokines in the detection of these memory T-cells.

## Results

### Baseline Characteristics

A total of 95 KTRs with a low antibody response after primary vaccination were enrolled in the RECOVAC repeated vaccination study at the Erasmus MC Rotterdam study site^20^; 88 participants met the eligibility criteria for booster vaccination and subsequent analyses. This cohort comprised of 31 individuals who received a single dose of mRNA-1273, 27 who received two doses of mRNA-1273, and 30 who received Ad26.COV2.S. In parallel, we compared the KTRs in our analysis to a healthcare worker (HCW) cohort; 30 individuals who were primed with mRNA-1273 and boosted with BNT162b2 were included in those analyses. Baseline characteristics are shown in Table 1. No significant differences were observed between the KTRs who received a different booster vaccination with regards to S1-specific binding antibody levels and T-cell responses at baseline (Table 1). The HCW cohort included a higher proportion of female participants, participants were younger, the interval since the last COVID-19 vaccination was shorter, and significantly higher levels of S1-specific binding antibodies at baseline were detected, compared to KTRs.

**Table 1:** Baseline characteristics of participants per assigned alternative vaccination strategy.

	KTRs			HCW	
Characteristic	1x mRNA-1273 (*N* = 31)	2x mRNA-1273 (*N* = 27)	Ad26.COV2-S (*N* = 30)	BNT162b2 (*N* = 30)	*p* value
Sex, no. (%)					0.006^b^
Male	22 (71)	15 (56)	20 (67)	9 (30)	
Female	9 (29)	12 (44)	10 (33)	21 (70)	
Age at time of first dose, (IQR) – yr	62.0 (51.0–69.0)	63 (55.0–67.5)	62.0 (54.0–70.0)	40.3 (34.8–52.6)	<0.0001^a^
BMI, (IQR) - kg/m^2^	25.9 (23.4–27.8)	25.0 (23.6–29.5)	25.9 (24.4–29.5)		0.77^a^
Number of comorbidities, (IQR)	1 (1 to 2)	1 (1 to 2)	1 (1 to 2)	-	0.76^a^
Comorbidities, no. (%)					
Hypertension	29 (94)	21 (78)	26 (87)	-	0.24^b^
Diabetes Mellitus	11 (35)	7 (26)	7 (23)	-	0.58^b^
History of coronary artery disease	2 (6)	3 (11)	5 (17)	-	0.45^b^
Heart failure	0 (0)	2 (7)	2 (7)	-	0.39^b^
Chronic lung disease	0 (0)	5 (19)	2 (7)	-	0.02^b^
History of malignancy^+^	5 (16)	6 (22)	0 (0)	-	0.02^b^
Auto-immune disease	1 (3)	1 (4)	0 (0)	-	0.76^b^
Creatanin (IQR) - umol/L	128.5 (96.3 to 163.8)	141.0 (109.0 to 175.0)	126.5 (112.5 to 170.3)	-	0.74^a^
Lymphocyte count, (IQR) - 10^9^/L	1.6 (1.3 to 2.0)	1.6 (1.4 to 2.0)	1.5 (1.3 to 1.9)	-	0.48^a^
Primary renal diagnosis, no. (%)					0.19^b^
Primary glomerulonephritis	7 (23)	2 (7)	2 (7)	-	
Familial/hereditary renal diseases	4 (13)	6 (22)	6 (20)	-	
Congenital diseases	5 (16)	0 (0)	0 (0)	-	
Vascular diseases	6 (19)	4 (15)	6 (20)	-	
Secondary glomerular/systemic disease	3 (10)	6 (22)	7 (23)	-	
Other	3 (10)	2 (7)	2 (7)	-	
Transplant characteristics					
First kidney transplant, no. (%)	28 (90)	22 (81)	25 (83)	-	0.31^b^
Time after last transplantation, (IQR) – yr	5.8 (3.3 to 10.3)	6.8 (2.0 to 11.5)	9.0 (4.3 to 14.6)	-	0.14^a^
Last transplant					
Living, no. (%)	21 (68)	21 (78)	23 (77)	-	0.71^a^
Number of immunosuppressive agents, (IQR)	2 (2 to 3)	2 (2 to 2)	2 (2 to 3)	-	0.22^a^
Immunosuppressive treatment, no. (%)					
Steroids	11 (35)	4 (15)	11 (37)	-	0.13^b^
Azathioprine	1 (3)	0 (0)	1 (3)	-	0.99^b^
Mycophenolate mofetil	29 (94)	25 (93)	29 (97)	-	0.86^b^
Calcineurin inhibitor	29 (94)	26 (96)	25 (83)	-	0.24^b^
Other	0 (0)	1 (4)	2 (7)	-	0.41^b^
Time since last SARS-CoV-2 vaccination, no. (%)	198.5 (198.0–200)	198.5 (196.3–223)	199.0 (198.0–225)	185.5 (170.0–194.0)	<0.0001^a^
Serological immune response prealternative vaccination					
S- specific binding antibodies, (IQR) - BAU/mL	4.27 (1.07–11.50)	1.65 (0.73–6.74)	1.26 (0.68–6.90)	729.0 (515.8–1422.5)	<0.0001^a^
S-specific binding antibody responders, no. (%)	10 (32)	6 (22)	6 (25)	30 (100)	<0.0001^b^
Cellular immune response prealternative vaccination					
IGRA IFN-γ, (IQR) - IU/mL	0.01 (0.00–0.02)	0.01 (0.00–0.01)	0.01 (0.00–0.12)	0.49 (0.23–1.08)	<0.0001^a^
IGRA IFN-γ responders, no. (%)	1 (3)	0 (0)	4 (13)	26 (87)	<0.0001^b^
IL-2, (IQR) - pg/mL*	2.20 (1.04–3.14)	1.60 (0.69 to 2.93)	2.26 (0.85–3.88)	-	0.50^a^
IL-4, (IQR) - pg/mL*	0.00 (0.00–0.79)	0.28 (0.00–0.87)	0.07 (0.00–1.36)	-	0.69^a^
IL-5, (IQR) - pg/mL*	0.26 (0.00–1.23)	0.82 (0.00–1.22)	0.55 (0.00v1.32)	-	0.87^a^
IL-9, (IQR) - pg/mL*	0.00 (0.00–0.24)	0.00 (0.00–1.26)	0.32 (0.00–1.29)	-	0.11^a^
IL-10, (IQR) - pg/mL*	0.44 (0.00–1.34)	0.56 (0.00–1.40)	0.50 (0.00– 1.18)	-	0.93^a^
IL-13, (IQR) - pg/mL*	1.53 (0.00–2.42)	0.64 (0.00–1.94)	0.00 (0.00 to 2.45)	-	0.65^a^
IL-17A, (IQR) - pg/mL*	0.00 (0.00–0.25)	0.00 (0.00–0.26)	0.13 (0.00–0.50)	-	0.21^a^
IL-17F, (IQR) - pg/mL*	0.04 (0.00–0.47)	0.00 (0.00–0.51)	0.13 (0.00–1.13)	-	0.45^a^
IL-22, (IQR) - pg/mL*	0.00 (0.00–0.19)	0.23 (0.00–0.92)	0.04 (0.00–0.62)	-	0.16^a^
TNF-α, (IQR) - pg/mL*	0.00 (0.00–3.84)	0.00 (0.00–3.03)	0.00 (0.00–4.32)	-	0.62^a^
IFN-γ, (IQR) - pg/mL*	1.46 (0.00–2.35)	0.63 (0.00–2.37)	1.63 (0.00–3.43)	-	0.38^a^

Values are number (percentage) for categorical variables and median [interquartile range] for continuous variables.

^a^*p* value based on non-parametric test (kruskal-wallis) test.

^b^*p* value based on fisher’s exact test.

*median ln(1+x) transformed SARS-CoV-2 specific T-cell response.

^+^Including melanomas, excluding all other skin malignancies

*BMI* body mass index, *IL* interleukin, *mo* month, *yr* year, *IGRA* interferon-gamma release assay.

### Vaccination boosts S1-specific antibodies in previously poorly responsive KTRs

**Fig. 1:**
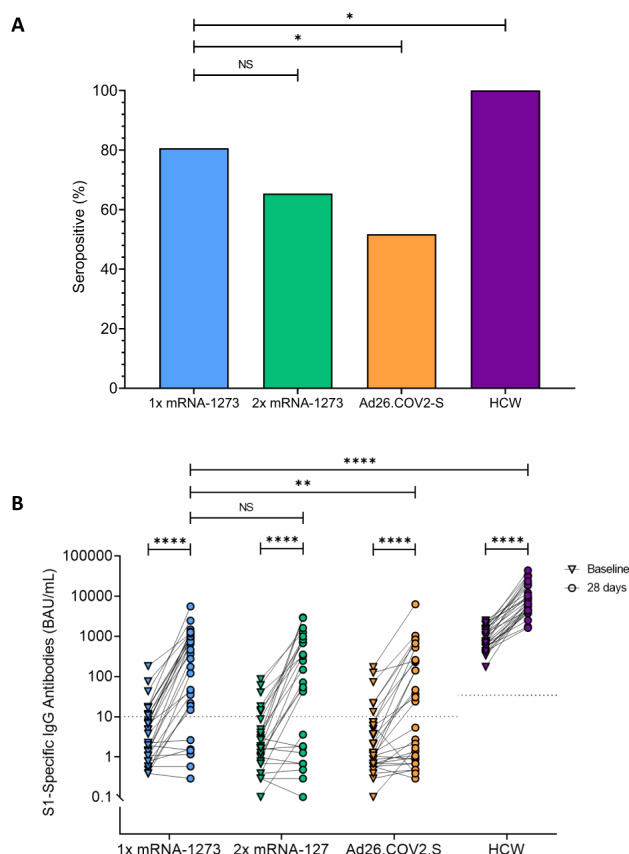
Serological response in alternative vaccination study groups. **A** Percentage of seroresponders per randomized alternative vaccination study group, including kidney transplant recipients (KTRs) and healthcare workers (HCWs), at 28 days after vaccination. Seroresponders for KTRs were defined as having a S1-specific IgG antibody level >10 BAU/mL, measured using a validated fluorescent bead-based multiplex immunoassay, and for HCWs >33.8 BAU/ml, as measured by the Liaison TrimericS IgG assay; *p* values were determined using the χ2 test. **B** SARS-CoV-2 S1-specific serum IgG antibody levels at baseline and 28 days after vaccination. Each participant is depicted by dots, and the dashed line represents the seropositivity thresholds. The p-values between groups were calculated using the Mann-Whitney U test, and the Wilcoxon Signed Rank test for intra-group comparisons. NS, no significance; *, *p* < 0.05; **, *p* < 0.01; ***, *p* < 0.001; ****, *p* < 0.0001.

S1-specific antibodies were measured before and 28 days after booster vaccination. Among KTRs, seropositivity rates at 28 days post booster vaccination increased from 32% to 80% after a single dose mRNA-1273, i.e. the reference vaccination regime, whilst seropositivity in participants who received a double dose of mRNA-1273 or a dose of Ad26.COV2.S increased from 22% to 65% and, from 20% to 51%, respectively (Fig. 1A). Among HCW, which were all seropositive at baseline, the seropositivity rate remained 100% after booster vaccination.

S1-specific antibody levels increased significantly in all three KTR groups as well as HCWs after booster vaccination (Fig. 1B). Although the majority of these KTRs produced antibodies after booster vaccination, levels were still significantly lower compared to HCWs (HCW versus KTRs who received a mRNA-1273: *p* < 0.0001).

### T-cell responses remained low after booster vaccination of KTRs

**Fig. 2:**
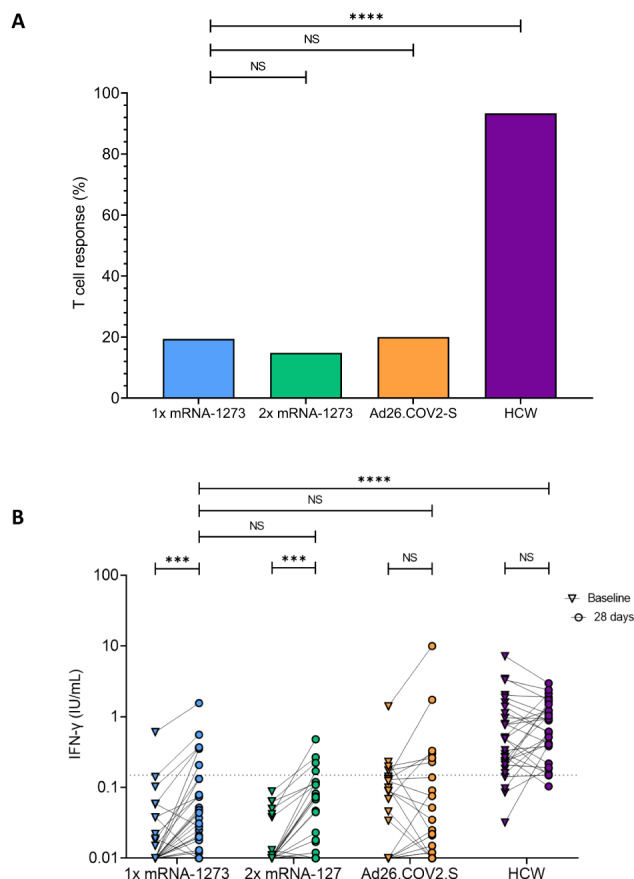
T-cell response in the alternative vaccination study groups. **A** Percentage of T-cell responders per randomized alternative vaccination study group at 28 days after vaccination. T-cell responders were defined as participants with an IFN-γ concentration >0.15 IU/mL; p-values were calculated using the χ2 test. **B** IFN-γ concentrations at baseline and 28 days after vaccination. Each participant is depicted by dots, with the dotted line indicating the cutoff value for T-cell response. The p-values between groups were calculated using the Mann-Whitney U test, and the Wilcoxon Signed Rank test for intra-group comparisons. NS, no significance; *, *p* < 0.05; **, *p* < 0.01; ***, *p* < 0.001; ****, *p* < 0.0001.

To quantify S-specific T-cell responses, secreted IFN-γ concentrations were measured in plasma after stimulation of whole blood with SARS-CoV-2 antigens before and 28 days after booster vaccination. The proportion of KTRs with measurable IFN-γ production was 29% after a mRNA-1273 booster, 33% after a double dose of mRNA-1273 booster, and 30% after a Ad26.COV2-S booster (Fig. 2A). For comparison, the proportion of HCWs with measurable IFN-γ production was 93% at 28 days post-booster.

Although T-cell responses in KTRs often remained below cut-off for positivity, IFN-γ concentrations significantly increased after single or double mRNA-1273 booster. Conversely, T-cell responses remained similar in Ad26.COV2.S-boosted KTRs (Fig. 2B). Of note, T-cell responses were significantly lower 28 days after booster vaccination compared to HCWs (HCW versus KTRs who received a mRNA-1273: *p* < 0.0001).

### Association between cytokine profiles and antibody production

**Fig. 3:**
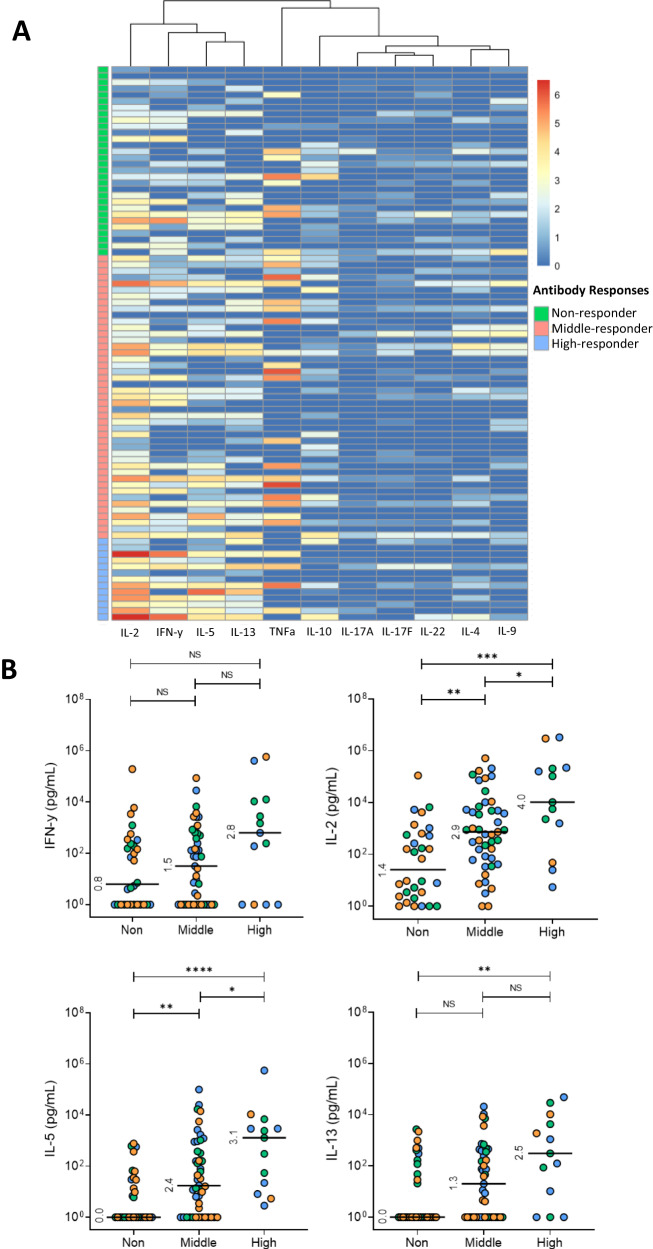
Antibody responder groups exhibit cytokine profiles after vaccination. **A** Heatmap illustrating ln_(x+1)_-transformed z-scores of SARS-CoV-2 specific T-cell cytokines based on S1-specific IgG antibody response groups at 28 days post-vaccination. The color scale (red-to-blue) represents ln_(x+1)_ T-cell cytokine values. The left banner of the heatmap indicates S1-specific IgG antibody response groups: non-responders, middle-responders, and high-responders. **B** Concentrations of the most differentially expressed T-cell cytokines for each antibody response group. Statistical analysis was performed using the Mann-Whitney U test to compare groups. Antibody response groups were defined based on the S1-specific IgG antibody levels at 28 days after vaccination: non-responders (S1-specific IgG <10 BAU/mL), middle-responders (S1-specific IgG 11-1000 BAU/mL), and high-responders (S1-specific IgG >1001 BAU/mL). NS, no significance; *, *p* < 0.05; **, *p* < 0.01; ***, *p* < 0.001; ****, *p* < 0.0001.

**Fig. 4:**
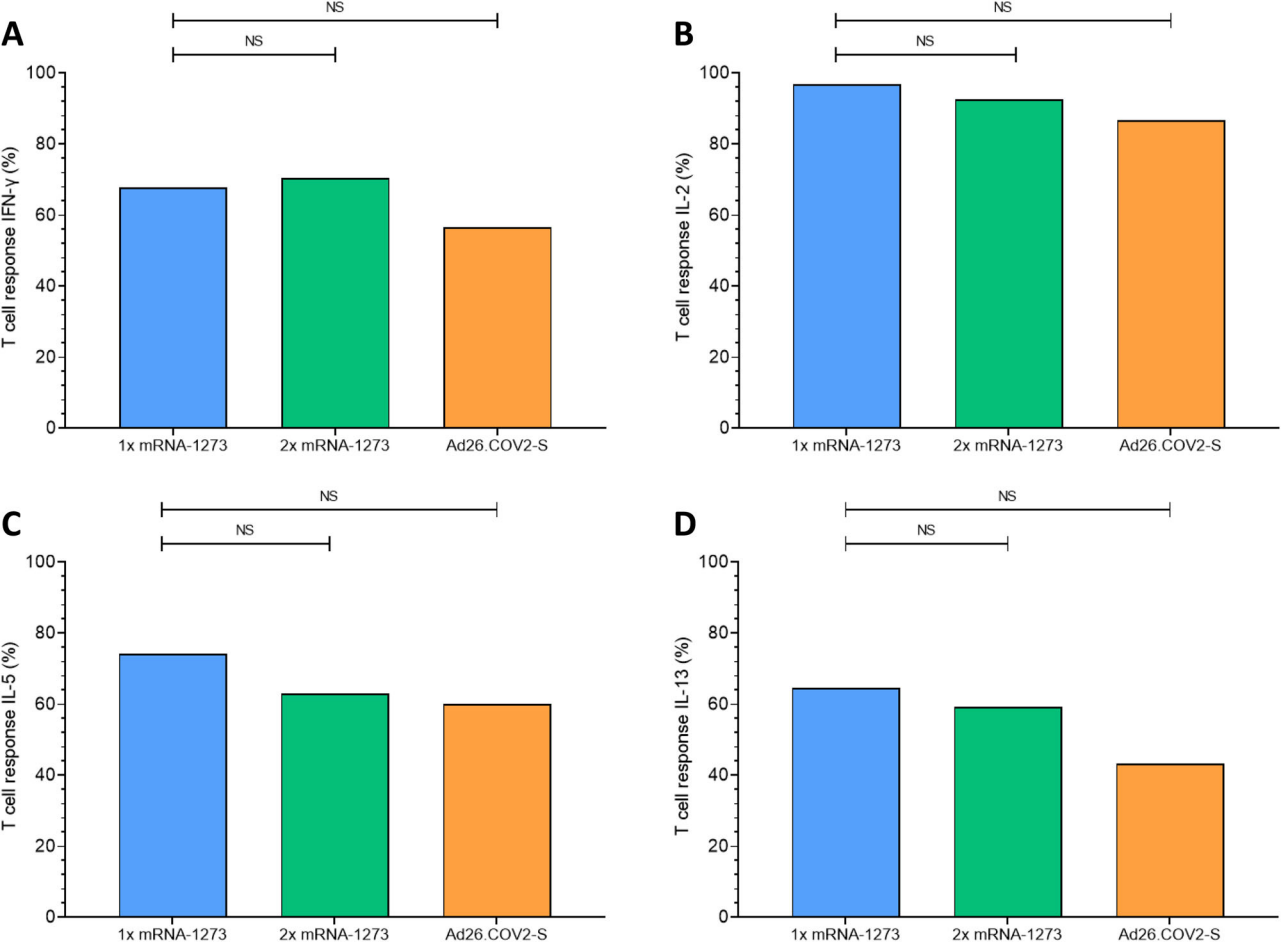
T-cell responders based on IFN-γ, IL-2, IL-5 or IL-13 in the alternative vaccination study groups. Percentage of T-cell responders per randomized alternative vaccination study group at 28 days after vaccination. **A** T-cell responders were defined as participants with an IFN-γ concentration >0.00 pg/mL. **B** T-cell responders were defined as participants with an IL-2 concentration >0.00 pg/mL. **C** T-cell responders were defined as participants with an IL-5 concentration >0.00 pg/mL. **D** T-cell responders were defined as participants with an IL-13 concentration > 0.00 pg/mL. *p* values were calculated using the χ2 test. NS, no significance; *p<0.05; **p<0.01; ***p<0.001; ****p<0.0001.

Since alternative booster vaccination strategies for KTRs increased antibody levels but not T-cell responses as measured by IFN-γ, we aimed to determine whether antibody responses after booster vaccination were correlated to various other T-cell-associated cytokines. To this end, we measured levels of 11 different cytokines in all 88 KTRs after booster vaccination. Next, KTRs were classified into three groups based on antibody response after booster vaccination, irrespective of their original vaccination group: non-responders (S1-specific IgG <10 BAU/mL), middle-responders (S1-specific IgG 11-1,000 BAU/mL), and high-responders (S1-specific IgG >1,001 BAU/mL). This analysis revealed notable differences in both the quantity and diversity of SARS-CoV-2-specific T-cell cytokines in different responder groups. While interleukin (IL)-17A, IL17-F, IL-22, IL-4 and IL-9 were hardly detected in any KTRs, IL-2, IFN-γ, IL-5, IL-13, TNFα and IL-10 was produced by the majority of KTRs. Visualization of cytokine levels in a heatmap revealed that IFN-γ, IL-2, IL-5, and IL-13 production were different between the antibody level groups (Fig. 3A). A closer examination of these cytokines showed that KTRs with high S1-specific IgG after booster vaccination had higher levels of IL-2, IL-5 and IL-13 compared to non-responders (Fig. 3B). A statistically significant increase in IL-2 and IL-5 concentrations was observed across the antibody responder groups (i.e., from non-responder, middle responder to higher responders). Although similar trends were observed for IL-13 and IFN-γ, this did not reach statistical significance. Differences were not driven by the vaccination groups (Fig. 4 and Supplemental Fig. 1).

### IL-2 and IL-5 as a sensitive readout parameter for T-cell responses in KTRs

**Fig. 5:**
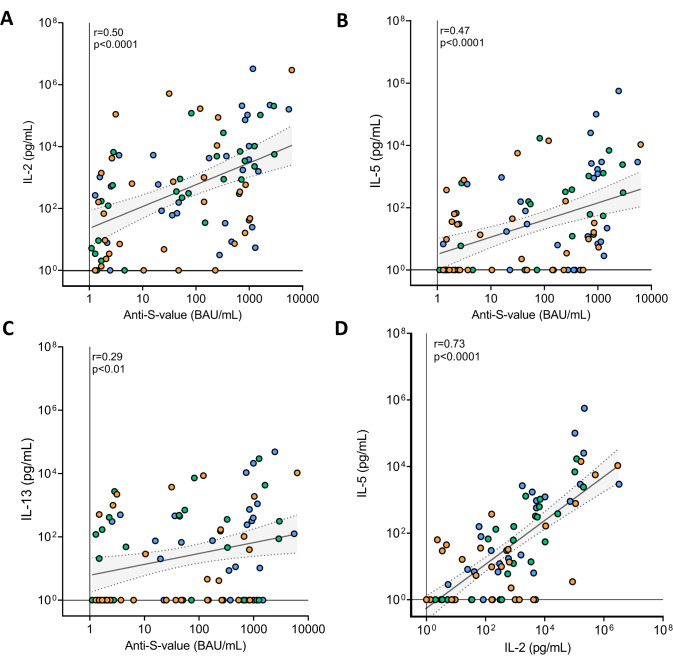
Correlation between cytokines and S1-specific IgG antibodies at 28 days after vaccination. **A** Correlation between IL-2 concentration and S1-specific IgG antibody levels (Spearman’s rank correlation coefficient 0.50; *p*<0.0001). The diagonal line represents the regression line on ln(x+1)-transformed data (beta coefficient 0.34; 95% CI 0.21 to 0.46). **B** IL-5 Spearman’s rank correlation coefficient 0.47; *p*<0.0001, beta coefficient 0.35; 95% CI 0.20 to 0.51. **C** IL-13 Spearman’s rank correlation coefficient 0.22; *p*<0.01, beta coefficient 0.22; 95% CI 0.06 to 0.39. **D** IL-2 concentration correlates with IL-5 concentrations 28 days after vaccination (Spearman’s rank correlation coefficient 0.73; *p*<0.0001). The diagonal line represents the regression line on ln(x+1)-transformed data (beta coefficient 0.66; 95% CI 0.54to 0.78). The gray shaded areas indicate the 95% CI of the best-fit line. Each symbol in the figure represents a participant.

Considering the minimal IFN-γ production post-booster in most vaccinated KTRs (Figs. 2 and 3), even in those with high levels of S1-specific antibodies (Fig. 1), we explored alternative T-cell associated cytokines as indicators for measuring T-cell responses in KTRs. Initial analyses revealed that IL-2, IL-5 and IL-13 could be potential candidates (Fig. 3A and B). When examining the percentage of T-cell responders, IL-2 emerged as the cytokine, which was produced by the greatest proportion of KTRs when compared to IFN-γ, IL-5 and IL-13 (Fig. 4). Next, we evaluated the correlations between IL-2, IL-5, and IL-13 levels with the S1-specific IgG levels (Fig. 5A-C). Notably, IL-2 and IL-5 levels were strongly correlated to the antibody response (r = 0.50, *p* < 0.001 and r = 0.48, *p* < 0.001, respectively), in contrast to the weaker correlations observed for IFN-γ and IL-13 (r = 0.23, *p* < 0.01, r = 0.27, *p* < 0.05, respectively). Interestingly, IL-2 and IL-5 exhibited similar kinetics, demonstrated by a robust correlation between the two (r = 0.73, *p* < 0.0001, Fig. 5D). None of the other measured cytokines demonstrated a correlation with S1-specific IgG levels (Supplemental Fig. 2).

## Discussion

In this study, we show that booster vaccination of KTRs with low serological responses after primary vaccination resulted in increased S-specific binding antibodies, as well as moderately enhanced IFN-γ T-cell responses. However, cytokine profiling suggest that IL-2 and IL-5 were more reliable markers to identify KTRs with a SARS-CoV-2-specific T-cell response, and that these markers were positively correlated to S1-specific antibody levels.

Despite the fact that booster vaccination increased immune responses, these were still inferior in KTRs when compared to the HCW group, a pattern consistent with prior research demonstrating KTRs enduring reduced responsiveness to booster doses^20,29–31^. It is important to note that our studied KTR and HCW groups were not age- and sex-matched and received different mRNA booster vaccines, and it is essential to acknowledge that many booster studies lack healthy controls for direct KTR comparisons^20,29–32^. In our study, HCWs and KTRs were matched based on their primary mRNA-1273 vaccination, but received different booster vaccines. Although mRNA-1273’s has been reported to lead to higher antibody levels compared to BNT162b2 when used as a priming regimen, these differences are less distinct when these vaccines are used as booster^33–36^. Nevertheless, mRNA-1273 remains more immunogenic following initial mRNA priming, underscoring KTRs’ reduced immunogenicity when compared to HCWs^36,37^. Although we report that mRNA-1273 was slightly more immunogenic as booster compared to Ad26.COV2.S in KTRs, these findings diverge from our larger study cohort, probably due to the lower number of participants in the sub-study presented here^20^. However, studies investigating the immune response to primary vaccination also reported lower immunogenicity of Ad.26.COV2.S compared to mRNA-based vaccines^16,38,39^.

The gold standard for assessing antigen-specific T-cell responses is the measurement of IFN-γ production after stimulation. Consequently, our initial assessment focused on the production of this cytokine using the IGRA assay. Notably, IFN-γ responses were significantly lower in KTRs compared to the HCWs; there was no significant effect based on the type of booster vaccination strategy employed. On average, only 31% of KTRs had a measurable IFN-γ response after booster vaccination, in sharp contrast to the serological responses, which showed that approximately 65% of KTRs had antibody responses. This outcome was unexpected, as prior research showed that solid organ transplant recipients and hematopoietic stem cell recipients develop T-cell responses even in the absence of antibody production^40,41^. To further characterize the T-cell response, we extended our analysis to measuring additional T-cell-associated cytokines, using a multiplex cytokine detection assay. In this analysis, we identified IL-2 and IL-5 to be highly correlated to antibody levels. This observation underscores the critical role of IL-2 in the antigen-specific T-cell response. Building on our previous study, in which we observed that a primary vaccination of KTRs led to predominant induction of IL-2-producing T-cells, rather than IFN-γ-producing T-cells, reaffirming IL-2 as a central cytokine of interest^14^. Importantly, in immunocompetent individuals, IL-2 exhibited a dominant influence over IFN-γ production in the induction of vaccine immunity across diverse vaccine types^42–44^. Despite identifying a potentially significant role for IL-5, the specific function of this cytokine in the context of vaccine research remains relatively unexplored.

The direct correlation between IL-2 (a typical Th1 cytokine) and IL-5 (a typical Th2 cytokine), and antibody titers, reinforces our prior discovery that showed the necessity of a balanced Th1 / Th2 cytokine profile induced by vaccination for robust antibody responses^14^. In KTRs, this equilibrium seems to be orchestrated by IL-2 and IL-5. This underscores the importance of measuring other cytokines than IFN-γ as potential biomarkers of T-cell responses when performing immunogenicity studies, particularly in this immunosuppressed patient population and raises questions regarding the suitability of the IFN-γ as a sole readout for accurately assessing cellular immune responses in KTRs^45^.

Our study has several limitations that warrant consideration. First, our investigation focused on cytokine profiles in KTRs with poor serological responses following primary vaccination. The applicability of our findings to good serologically responders is unknown, emphasizing the need for caution when generalizing these results to a broader population. Second, it is important to acknowledge the distinctive immunological context of our KTRs. The individuals receive immunosuppressive therapy, a known factor that significantly suppresses cytokine responses. As a result, our findings may not readily extrapolate to immunocompetent individuals, for whom T-cell responses occur under different conditions and other cytokines could be more important^46,47^. Third, our study lacks data regarding breakthrough infections within these groups, which would have provided valuable insights into the potential correlates of protection, as well as the role of T-cell responses in this context. Finally, our assessment was conducted exclusively at 28 days after booster vaccination. The long-term development and durability of these responses is subject for future evaluation.

In conclusion, our study provides insight into SARS-CoV-2-specific T-cell responses following booster vaccinations in KTRs who initially exhibited poor responsiveness. It emphasizes the importance of the induction of balanced T-cell responses in KTRs and underlines the correlation between specific T-cell cytokines and antibody production. These findings suggests that broader examination of T-cell cytokines could be a promising approach for assessing immune responses to vaccines.

## Methods

### Participants and alternative COVID-19 booster vaccination

Samples were collected from 88 participants enrolled at the Erasmus MC Rotterdam in an open-label randomized controlled trial evaluating alternative booster vaccination strategies for KTRs^20^. This trial was conducted as part of the multicenter Dutch Renal patients COVID-19 VACcination (RECOVAC) study^12^. Ethical approval for the RECOVAC study was granted by the Dutch Central Committee on Research Involving Human Subjects (CCMO, NL78963.042.21) and the institutional review board of the Erasmus MC Rotterdam. The study was registered on clinicaltrials.gov (NCT05030974). Written informed consent was obtained from KTRs who did not seroconvert after receiving two doses of the mRNA-1273 COVID-19 vaccine and were enrolled and randomized, as outlined in our prior research^20^. Whole blood samples were collected both before (baseline) and 28 days after vaccination, samples were processed within 12 hours. Antibody and T-cell responses were compared to a convenience control cohort consisting of HCWs. For this study, we analyzed 30 HCWs who were primed with two shots of mRNA-1273, followed by boosting with BNT162bv2. Whole blood samples were collected before (baseline) and 28 days after third vaccination, aligning with the timing of KTR sample collections. Ethical approval for the HCW study was granted by the institutional review board of the Erasmus MC (medical ethical committee, MEC-2020-0264).

### SARS-CoV-2 S1-specific IgG binding antibodies

For KTRs, SARS-CoV-2 S1-specific IgG binding antibodies were measured in serum samples using a validated fluorescent bead-based multiplex immunoassay. The assay’s specificity and sensitivity have been previously determined and described, achieving values of 99.7% and 91.6%, respectively^48,49^. The antibody levels were expressed as international binding antibody units per mL (BAU/mL). Based on a Receiver Operator Curve (ROC) analysis, patients were classified as either seropositive or seronegative, with the threshold for seropositivity defined as a S1-specific IgG concentration of ≥10 BAU/mL^48,50^. For HCWs, S1-specific IgG were measured by Liaison SARS-CoV-2 TrimericS IgG assay (DiaSorin, Italy), with a lower limit of detection of 4.81 BAU/mL and a cut-off for positivity at 33.8 BAU/mL. The assay was performed following the manufacturer’s instructions.

### SARS-CoV-2 specific T-cell cytokine responses

For KTRs and HCWs, SARS-CoV-2-specific T-cell responses were measured using the commercially available IFN-γ Release Assay (IGRA, QuantiFERON, QIAGEN, Hilden, Germany). Heparinized whole blood samples were used, following the methodology as described previously^14^. In brief, heparinized whole blood was incubated with SARS-CoV-2 antigen tubes containing overlapping peptides representing the S protein, stimulating both CD4+ and CD8 + T-cells (Ag2), for 20-24 hours at 37°C. After incubation, plasma was collected and frozen for subsequent analysis. A validated ELISA (QIAGEN) was performed to quantify IFN-γ levels; results were expressed in IU/mL. The cut-off value for test positivity in IFN-γ production was 0.15 IU/mL, according to manufacturer’s instructions.

For KTRs, an additional human Th cytokine panel kit (LEGENDplex, Biolegend, CA, USA) was used to quantify cytokines present in the plasma of SARS-CoV-2 S protein antigen-stimulated whole blood samples, as obtained above for IGRA^20^. This panel included interleukin (IL)-2, IL-4, IL-5, IL-6, IL-9, IL-10, IL-13, IL-17A, IL-17F, IL-22, IFN-γ, and TNF-α. Plasma samples were thawed on ice, centrifuged, and twofold dilutions were prepared. The diluted samples were incubated with monoclonal capture antibody-coated beads for 2 hours. Subsequently, the beads were washed and incubated with biotin-labeled detection antibodies for one hour, followed by incubation with streptavidin-PE for 30 minutes. After staining, the beads were analyzed by flow cytometry using a BD FACSCanto™ II with BD FACSDiva™ software (BD Bioscience, NJ, USA). The acquired data were analyzed with LEGENDplex V8.0 software (BioLegend). The quantity of each cytokine was calculated based on the intensity of the streptavidin-PE signal and a freshly prepared standard curve. The results were expressed in picogram cytokine/mL (pg/mL) after subtracting the NIL control value. In cases where the subtraction resulted in a negative value, the value was set at 0 pg/mL. As an internal quality control for the cytokine measurements, we performed Spearman’s correlation analysis on the IFN-γ concentrations of the same samples measured by both ELISA (data presented in the original publication^20^) and multiplex bead assay, and found that these were strongly correlated (Supplemental Fig. 3).

### Statistical analysis

First, we presented the baseline characteristics of each vaccination group within the KTRs and HCWs. Categorical variables were reported as numbers (percentages), and Fisher’s exact test was utilized to assess group differences. Continuous variables were presented as median (interquartile ranges), and differences between medians among groups were evaluated using the Kruskal-Wallis test for the alternative vaccination strategies. Second, the levels of the S1-specific binding IgG antibodies and T-cell cytokines produced were reported. Differences between groups were assessed using Mann Whitney-U test or Pearson Chi-square test, depending on data type and distribution. Additionally, the Wilcoxon Signed Rank test was employed to investigate differences within groups. Third, the cytokine values obtained 28 days after the second vaccination were ln(x + 1)-transformed. KTRs were categorized into three antibody responder categories based on the antibody titers at 28 days after vaccination: non-responders (<10 BAU/mL), middle-responders (11–1000 BAU/mL), and high-responders (1,001-6,303 BAU/mL). A heatmap was generated using the R package pheatmap (V1.0.12) to visualize the cytokine responses across the antibody responder categories. Differences in cytokine levels between the antibody responder categories were assessed using Mann Whitney-U test. Finally, Spearman’s correlation coefficient was calculated to explore relationships between S1-specific binding antibodies and T-cell cytokines. Statistical analyses were conducted using GraphPad Prism software version 9.1.2 and Rstudio software (version 4.0.5). A *p* value < 0.05 was considered statistically significant.

## Supplementary information

Supplementary information

## Data Availability

All data used to support the findings of this study are available from the corresponding author upon reasonable request.

## Code availability

Please contact the corresponding author to obtain the R code used in this study
