# Supplementary material for: Revealing the significance of IL-2 and IL-5 in SARS-CoV-2-specific T-cell responses in kidney transplant recipients
Source: Npj Viruses. 2024 Feb 14;2:7. doi: 10.1038/s44298-024-00015-7 (PMC11702830; doi:10.1038/s44298-024-00015-7)
Supplement: Supplementary file 1 — Supplementary information [file 44298_2024_15_MOESM1_ESM.pdf]

Supplementary File for

Revealing the Significance of IL-2 and IL-5 in SARS-CoV-2-specific T-cell responses in Kidney Transplant Recipients

Yvette den Hartog[1], S. Reshwan K. Malahe[1], Wim J.R. Rietdijk[2], Marjolein Dieterich[1], Lennert Gommers[3], Debbie van Baarle[4,5], Dimitri A. Diavatopoulos[6,7], A. Lianne Messchendorp[8], Renate G. van der Molen[6], Ester B.M. Remmerswaal[9], Frederike J. Bemelman[10], Marcia M.l. Kho[1], Corine H. GeurtsvanKessel[3], Marion P.G. Koopmans[3], Ron T. Gansevoort[8], Luuk B. Hilbrands[11], Jan-Stephan Sanders[8], Marlies E.J. Reinders[1], Carla C. Baan[1], Rory D. de Vries[3#] on behalf of RECOVAC Consortium*

[1] Department of Internal Medicine, Nephrology and Transplantation, Erasmus MC Transplant Institute, University Medical Center Rotterdam, The Netherlands
[2] Department of Hospital Pharmacy, Erasmus MC, University Medical Center Rotterdam, The Netherlands
[3] Department of Viroscience, Erasmus MC, University Medical Center Rotterdam, The Netherlands
[4] Department of Medical Microbiology and Infection Prevention, University Medical Center Groningen, Groningen, The Netherlands
[5] Center for Infectious Disease Control, National Institute for Public Health and the Environment, Bilthoven, The Netherlands
[6] Department of Laboratory Medicine, Laboratory of Medical Immunology, Radboud University Medical Center Nijmegen, Nijmegen, The Netherlands
[7] Radboud Center for Infectious Diseases, Radboud University Medical Center Nijmegen, Nijmegen, The Netherlands
[8] Department of Internal Medicine, Division of Nephrology, University of Groningen, University Medical Center Groningen, Groningen, the Netherlands
[9] Department of Experimental Immunology, Amsterdam Infection and Immunity Institute, Amsterdam UMC, University of Amsterdam, Amsterdam, The Netherlands
[10] Renal Transplant Unit, Amsterdam UMC, University of Amsterdam, Amsterdam, The Netherlands
[11] Department of Nephrology, Radboud University Medical Center, The Netherlands

*Lists of members and their affiliations appears in the Supplementary Information

[#] Corresponding author: Rory D. de Vries; **e-mail:** r.d.devries@erasmusmc.nl; **telephone:** +31107044099; **address:** Dr. Molewaterplein 40, 3015 GD, Rotterdam, the Netherlands.

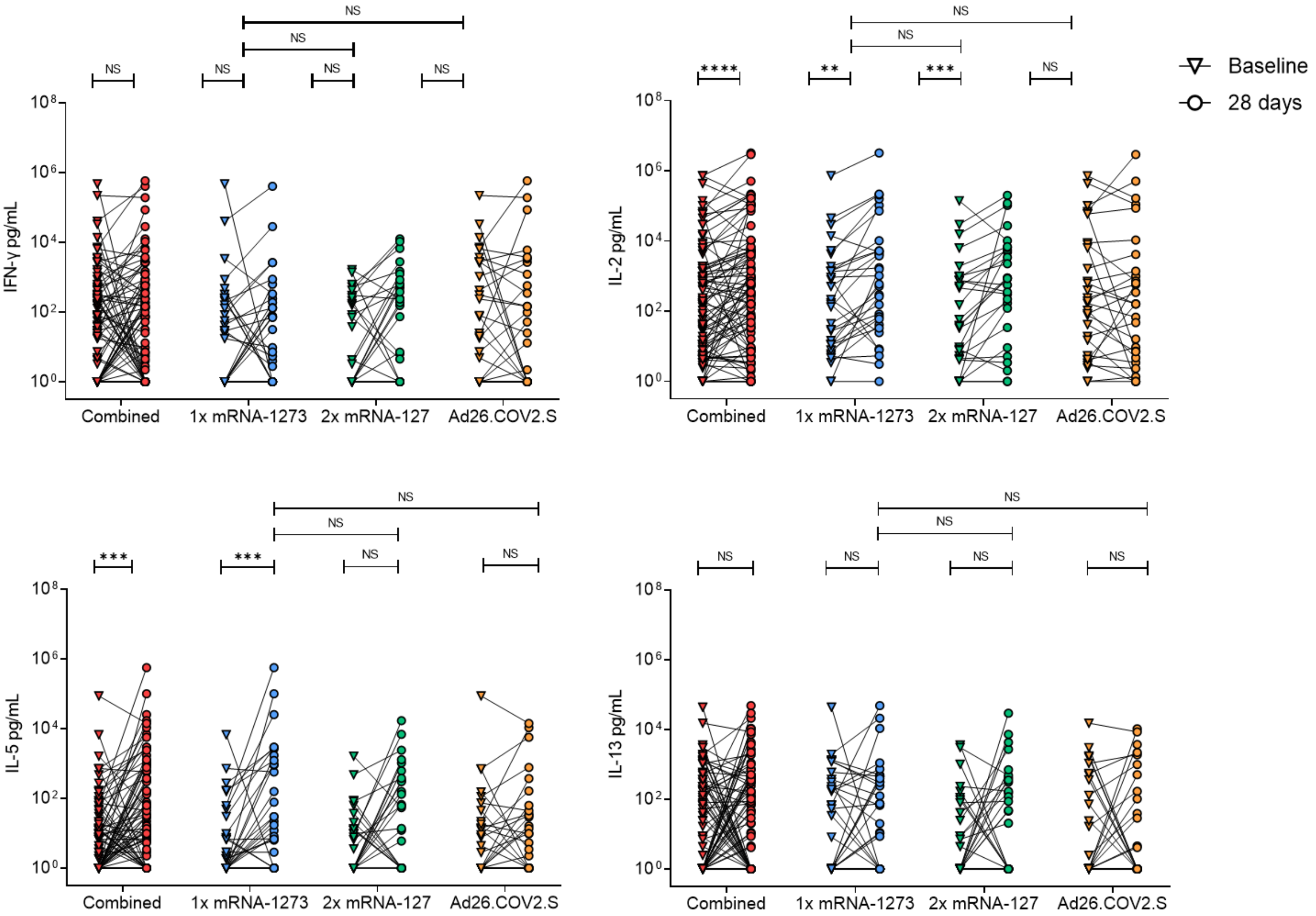

Supplemental Figure 1 T-cell cytokines measured by LEGENDPLEX in the alternative vaccination study groups. SARS-CoV-2 specific T-cell cytokines at baseline and 28 days after vaccination. The p-values between groups were calculated using the Mann-Whitney U test, and the Wilcoxon Signed Rank for intra-group comparisons. NS, no significance; *, p<0.05; **, p<0.01; ***, p<0.001; ****, p<0.0001.

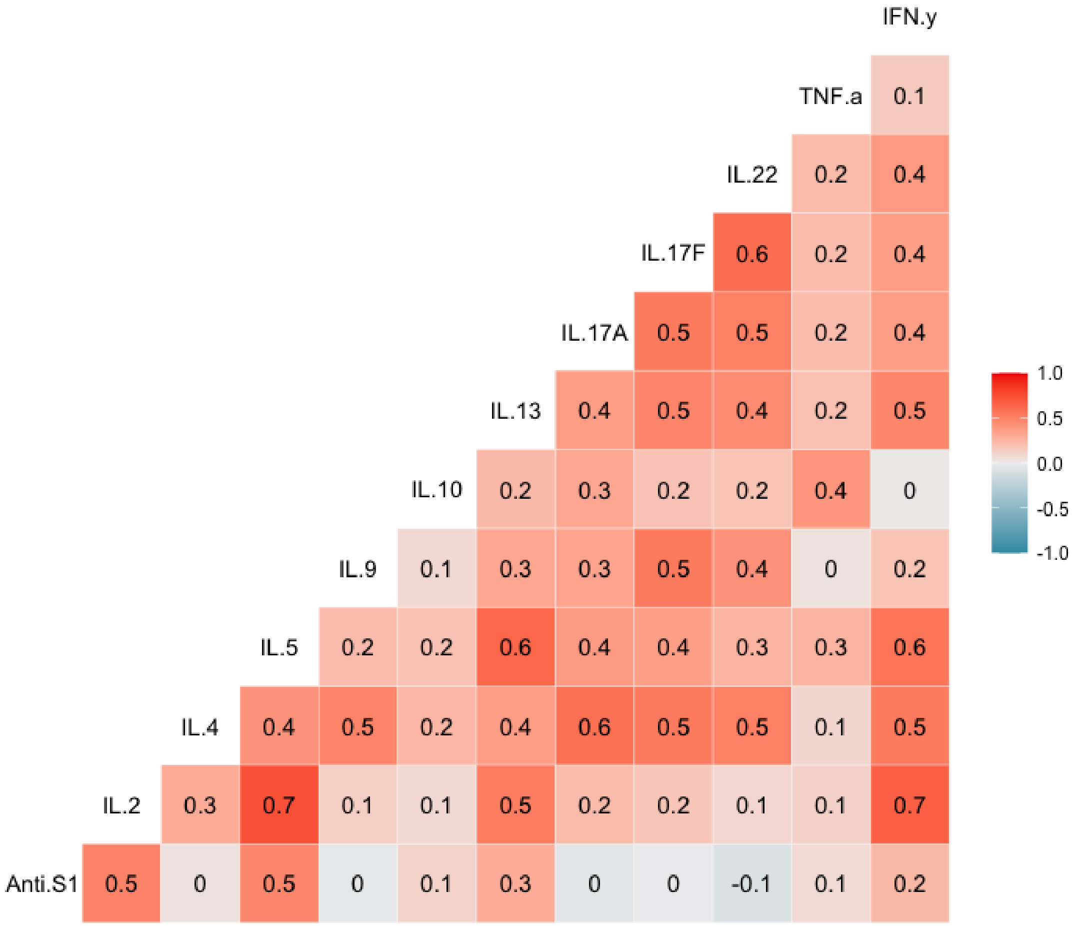

Supplemental Figure 2 Correlation matrix T-cell cytokines and S1-specifc IgG antibodies at 28 days post-vaccination. Spearman’s rank correaltions between the concentration of cytokines produced by SARS-CoV-2 specific T-cells and S1-specifc IgG antibody levels. The correlations are caluculated on ln(x+1)-transformed data.

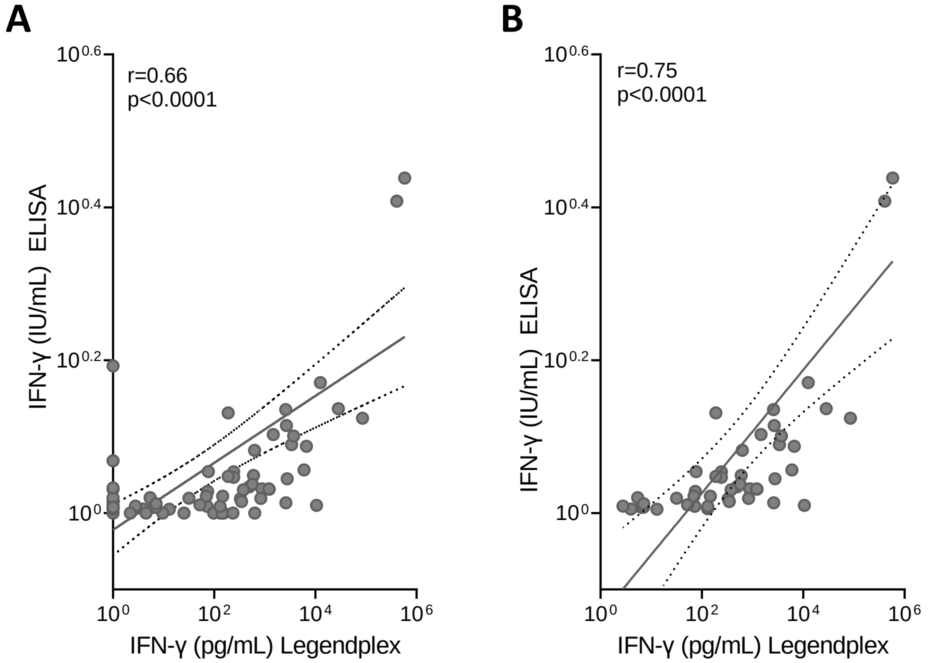

Supplemental Figure 3 Correlation of Legendplex and ELISA SARS-CoV-2 specific IFN-γ ex vivo stimulation. A) Legendplex measured IFN-γ levels correlated with ELISA measured IFN-γ levels, 28 days after third vaccination (Spearman's rank correlation coefficient 0.66; p<0.0001). Diagonal line represents the regression line on ln(x+1)-transformed concentrations in Legendplex and ELISA (n=88) data (beta coefficient 0.04; 95% CI 0.0.03 to 0.06). The 95% CI limits of the best-fit line are presented as dotted lines. B) Legendplex measured IFN-γ levels correlated with ELISA measured IFN-γ levels, 28 days after third vaccination (Spearman's rank correlation coefficient 0.75; p<0.0001). Diagonal continuous line represents the regression line on ln(x+1)- transformed responder in both Legendplex and ELISA (n=47) data (beta coefficient 0.08; 95% CI 0.05 to 0.11). The 95% CI limits of the best-fit line are presented as dotted lines. Each symbol represents a participant.
